# Students managing work and study role boundaries: a person-centred approach

**DOI:** 10.3389/fpsyg.2023.1116031

**Published:** 2023-06-20

**Authors:** Peter A. Creed, Michelle Hood, Andrea Bialocerkowski, M. Anthony Machin, Paula Brough, Sujin Kim, Sonya Winterbotham, Lindsay Eastgate

**Affiliations:** ^1^School of Applied Psychology, Griffith University, Southport, QLD, Australia; ^2^Centre for Work, Organisation, and Wellbeing, Griffith University, Nathan, QLD, Australia; ^3^Office of the Pro Vice Chancellor, Griffith Health, Griffith University, Southport, QLD, Australia; ^4^School of Psychology and Wellbeing, University of Southern Queensland, Toowoomba, QLD, Australia

**Keywords:** role boundary management, work flexibility, work-study demands, work-study conflict, study burnout, future employability

## Abstract

To cope with demands of working while studying, students must structure the boundaries between these roles (e.g., integrate or segment them) to suit their preferences and circumstances. However, students differ on how well they do this, and we do not yet understand the factors that contribute to managing work and study well. We sought to determine if different student groups existed and if the groups reported different work, study, and wellbeing outcomes. Using latent profile analysis and assessing work-study boundary congruence and flexibility (*N* = 808; 76% female; *M*_Age_ 19.6 years), we identified four groups of (a) “balanced” (65.4%; with moderate boundary congruence and flexibility); (b) “high work congruence and flexibility” (17.5%; working arrangements supportive of study role); (c) “low work congruence and flexibility” (9.7%; unsupportive workplace arrangements); and (d) “low study congruence” (7.3%; study arrangements unsupportive of work role). These groups reported different work/study demands, role conflict, study burnout, and perceived employability, with “balanced” and “high work congruence and flexibility” groups scoring more positively and “low work congruence and flexibility” and “low study congruence” groups scoring more negatively. Results supported that different student groups existed, and these will need different supports to manage their multiple role responsibilities.

## 1. Introduction

Around 80% of students enrolled in Australian universities juggle work with study (Australian Bureau of Statistics, 2021), which is consistent with statistics reported in other developed countries, such as the United States (85%; HSBC, 2018). By-and-large, working students are employed in precarious jobs (Campbell and Price, 2016), characterised by poor conditions, irregular hours, low pay, insecurity, and little regulatory protection (Vosko, 2011; Creed et al., 2020a,b). They work to generate discretionary spending and cover living expenses and tuition fees (HSBC, 2018). Those who can manage these two important, but often challenging and competing roles (e.g., for time and energy), report better study engagement, performance, and wellbeing (Chu et al., 2021a,b). Our aim was to determine whether different clusters (groups, types) could be identified in the seemingly heterogeneous population of working students based on differences in congruence and flexibility in these two roles.

Understanding how students manage the boundaries, or interface, between work and study roles can help explain how they meet their work and study obligations and account for study, career, and wellbeing outcomes (Kreiner et al., 2009). Variable-centred research has shown that work-study boundary congruence (i.e., attained fit or correspondence between work and study roles) is associated with better psychological wellbeing and more study engagement in working students (Chu et al., 2021b). However, understanding how different types of students manage their role boundaries, and how these different patterns might relate to important student outcomes, can inform those who assist students, such as counsellors and education providers. As a result, we took a person-centred approach.

The study was informed by general interactionist and person-environment fit theories (Muchinsky and Monahan, 1987; Van Vianen, 2018), which state that behavioural variability occurs because of the interaction between the person and the environment, that the person will seek out environments that best suit them, that better suited environments are related to better outcomes, and that individuals will attempt to manage their environment and their responses to it to improve fit. We expected to identify different patterns of behaviour in working students depending on their work-study preferences, the preferences of others, situational affordances, and their capacity to manage the work-study interface to their advantage. While testing for different groupings based on membership patterns is an exploratory methodology (Spurk et al., 2020), previous research in the vocational psychology area has identified clusters based on career development variables in both working adults (for review, see Spurk et al., 2020) and university students (Araújo et al., 2019).

We operationalised person/environmental and boundary management constructs using two sets of variables: (a) perceived work and study boundary congruence and (b) the capacity to manage the person-fit interaction (i.e., work and study flexibility-ability and flexibility-willingness, reflecting the individual’s capacity to influence fit and the motivation to do so; Matthews and Barnes-Farrell, 2010). Additionally, we aimed to validate any patterns identified by examining differences among them on five variables important to working students: work and study demands, role conflict, study burnout, and students’ view of their future employability.

Boundary congruence theory proposes that an individual’s multiple roles, such as work and study roles, are separated by cognitive, physical, and behavioural boundaries, and that people strive to more-or-less segment or integrate these roles depending on their boundary preferences and the constraints imposed by the environment (e.g., job demands, study needs). Further, individuals strive to increase role congruence (i.e., to maximise integration or segmentation), and perform better and feel more satisfied when they do so (Ashforth et al., 2000; Chen et al., 2009; Kreiner et al., 2009). For example, those able to generate more work-study congruence (e.g., by co-ordinating work and study schedules) should be able to meet their work and study responsibilities better and be more satisfied with their work and study arrangements. Chu et al. (2020) tested these relationships and showed that that higher work-study boundary congruence was related to better wellbeing and more study engagement in working students. In contrast, boundary incongruence (e.g., less integration than desired) generates conflict between roles and reduces capacity to meet responsibilities in both (Brough and O’Driscoll, 2015). For working students, this can make it more difficult to study as well as work (Wan et al., 2022). Thus, boundary congruence can be considered to be the level to which individuals have shaped their role boundary interface to suit their own preferences and the wishes of others, such as job supervisors and study colleagues (Chu et al., 2018).

The capacity to generate boundary congruence is influenced by the person’s social and physical environments, over which the individual does not have full control. This is reflected in boundary flexibility-ability, or the “individual’s perception of personal and situational constraints that affect boundary management” (p. 330), and boundary flexibility-willingness, the “motivationally oriented individual difference variable that contributes to actual levels of domain segmentation-integration” achieved (Matthews and Barnes-Farrell, 2010; p. 332). For example, working students will identify opportunities to adjust the overlap between study and work roles (i.e., increase or decrease work-study integration or segmentation – reflecting boundary flexibility-ability) and will differ on their motivation to implement such adjustments (i.e., reflecting boundary flexibility-willingness). Previous studies have shown that a capacity to ease movement between role domains is a resource for the person (Ashforth et al., 2000; Matthews and Barnes-Farrell, 2010; Winkel and Clayton, 2010), while in the career area, Creed et al. (2022) showed that both flexibility-ability and flexibility willingness were related to less burnout and greater engagement in working students.

From the above, we expected that different groups of working students would show different patterns, or styles, of functioning based on perceived boundary congruence and the role interface management strategies regarding boundary flexibility and willingness, reflecting both individual and environmental factors, consistent with interactionist and fit theories (Muchinsky and Monahan, 1987; Van Vianen, 2018) and boundary theory (Chen et al., 2009). To test this proposal, we used latent profile analysis (LPA), which is a person-centred method suitable for identifying distinct groupings of individuals based on different configurations of person- and/or environmental-level characteristics. Person-centred approaches like LPA are important research methods as they supplement variable-level methodologies, which test relationships among individual variables (Spurk et al., 2020).

We found no studies that had tested for sub-populations of working students based on boundary management constructs, such as boundary congruence and management strategies. In the career area, person-centred approaches have been applied to assess groupings based on work motivation, job crafting, organisational commitment, and career adaptability (for review, see Spurk et al., 2020); while one study in the work-family area tested for boundary management styles and their relationship to work and family functioning, finding that low control styles were associated with poorer work and family outcomes (Kossek et al., 2012).

Because of the exploratory nature of LPA, we developed a non-specific hypothesis, but one based on an examination of the literature regarding boundary management and with reference to relevant theories. LPA enables exploration of both quantitative (i.e., differences on profile scores) and qualitive profile differences (i.e., differences on overall profile shapes; e.g., profiles might be parallel or non-parallel; Marsh et al., 2009). Quantitative differences might suggest that different groups would benefit from different types of skills development, whereas qualitative differences might indicate that all groups could benefit from the same training, but at different levels. Both score and shape differences support validity that the profiles identified reflect true population differences (Spurk et al., 2020). The latent profile analysis hypothesis was:

*Hypothesis 1*: Different profiles exist for working students that reflect person and environmental characteristics implicit in perceptions of boundary congruence and flexibility (ability and willingness).

In addition, we aimed to test how any resultant profiles might differ on important outcome variables for working students. These outcomes were work and study demands, role conflict, study burnout, and perceived future employability. Testing these relationships would provide support for convergent validity for the different profiles identified, while allowing insight into how and when interventions might be required for students with different styles of boundary management between work and study.

*Work and study demands* refer to the person and situational challenges encountered on-the-job and at university that are associated with some level of personal cost, such as sapping energy and depleting personal resources (Demerouti et al., 2001). Workplace demands are related to poorer academic performance, satisfaction (Taylor et al., 2020; Wan et al., 2022) and wellbeing (Carney et al., 2005; Mounsey et al., 2013). Similarly, excessive study demands are related to more negative outcomes in students, including poorer mental health (Salmela-Aro and Upadyaya, 2014) and less academic engagement (Cilliers et al., 2018).

*Role (work-study) conflict* occurs when participation in one role negatively affects performance and functioning in another (Greenhaus and Beutell, 1985; Greenhaus and Powell, 2006). For example, students’ over-commitment at work (e.g., working long hours) conflicts with study by reducing the time and effort available. Work-study role conflict is associated with more negative feelings about study (Creed et al., 2015), greater mental distress (Waterhouse et al., 2020), less study effort, and poorer grades (Meeuwisse et al., 2017).

*Study burnout* refers to the physical and mental exhaustion and cynical attitude that develops towards study, which results from ongoing exposure to demanding and emotionally draining student situations (Marôco and Campos, 2012). University students are especially susceptible to burnout as they must simultaneously managing academic performance, work and survival demands, and numerous developmental challenges (e.g., around identity and relationship development). In working students, burnout is related to poorer outcomes, such as less study engagement (Barratt and Duran, 2021) and reduced academic (Galbraith and Merrill, 2015) and job performance (Vîrgă et al., 2019).

Finally, student *perceived future employability* is their view of how employable they will be after completing formal education (Gunawan et al., 2019). Higher perceptions of future employability are related to positive outcomes, including holding higher career aspirations, engaging in more career planning, exerting more effort to career progression, having stronger study commitment and academic performance, and experiencing less career indecision and distress (Gunawan et al., 2019, 2021; Creed et al., 2020a,b).

Importantly, all outcomes have been shown to be related to boundary management practices (for review, see Eastgate et al., 2021). For example, work-study boundary permeability (i.e., how easy it is for role responsibilities to cross role boundaries) is associated with more work and study role overload and greater work and study demands (Wan et al., 2022); work-study boundary segmentation (i.e., strength of boundary separation between roles) is related to less work-study conflict and more enrichment (van Steenbergen et al., 2018); and work-study boundary congruence is related to better wellbeing and higher levels of perceived future employability (Chu et al., 2021a,b). From a theoretical perspective (e.g., job-demands model; Owen et al., 2018), challenging boundary management experiences and negative boundary management outcomes drain personal resources and reduce energy and enthusiasm that could be applied to work and study activities.

We generated broad expectations related to profile-outcome relationships, although these were informed by previous studies and theory. We anticipated that profiles that were higher on boundary congruence and/or role interface management strategies (i.e., boundary flexibility capacity and willingness) would return more positive scores on the outcomes (e.g., perceiving fewer work-study demands, less work-study conflict, less burnout, and higher perceived employability), vis-à-vis those lower on congruence and management strategies.

*Hypothesis 2*: Profiles higher on work-study boundary congruence and role interface management strategies show lower workplace demands, study demands, and study burnout, and higher perceived future employability.

To date, it has not been demonstrated that there are distinct patterns of boundary management styles in working students and, if there are, whether they relate to variables that are important for students’ present and future functioning and achievement. We contribute to the literature in this area by applying LPA to explore working student boundary profiles based on boundary congruence, flexibility-ability, and flexibility-willingness. Boundary management success is reflected in students’ own preferences (i.e., for integration/ segmentation), the preferences of others, situational affordances, and students’ capacity and willingness to influence the work-study boundary interface for their own benefit. Second, we tested for relationships between identified profiles and variables of work-study demands, role conflict, study burnout, and perceived future employability. Identifying different groupings of working students, and the way they relate to career outcomes, can inform interventions to assist those struggling to manage work and study responsibilities.

## 2. Materials and methods

### 2.1. Participants

Participants were 808 undergraduate students recruited from two Australian universities (75.9% female; mean age 19.63 years, *SD* = 2.24, range 17–25 years). Almost all were domestic students, with a small proportion being international students studying in Australia. They were enrolled in foundation year courses in their respective universities that catered for students from a wide range of study program (e.g., business, counselling, nutrition, education, psychology, communications, medicine, and architecture); thus, the sample was quite heterogeneous for study interests and ability. All were working, as this was the inclusion criterion (mean hours worked per week = 18.76, *SD* = 9.56), mostly in casual or ongoing, part-time jobs in the service and leisure industries. For financial situation, 31% indicated *living comfortably on present income*, 46% were *managing*, 19% were *finding it difficult*, and 5% were *finding it very difficult* (mean 1.98, *SD* = 0.82; European Social Survey, 2010).

### 2.2. Measures

Participants answered questions using 6-point Likert-like responses (1 *strongly disagree* to 6 *strongly agree*), unless otherwise noted. Item scores were summed to generate totals, with higher total scores reflecting higher levels of the construct being measured. Internal reliability coefficients were calculated using Cronbach’s alpha formula and construct validity was supported by testing all scales together in a confirmatory factor analysis (CFA).

*Work congruence*. The 4-item Occupational Congruence Subscale from the Work-Study Congruence Scale (Chu et al., 2018) assesses the degree to which supervisors and colleagues in the workplace support work flexibility to meet student study demands (e.g., “My work supervisor will consider my study commitments when setting work rosters”). Chu et al. (2018) reported an Cronbach alpha of 0.92 and supported validity by finding expected correlations with other role conflict constructs. With the current sample, alpha (α) was 0.85.

*Study congruence*. The 4-item University Demands and Resources Subscale from the Work-Study Congruence Scale (Chu et al., 2018) assesses participants’ level of flexibility provided by their study program (e.g., “The availability of study or lecture material online makes it easier for me to work ad study”). Chu et al. (2018) reported α = 0.83 and support for validity from a negative relationship with work-study incongruence. Our α was = 0.74.

*Work and study flexibility and ability*. We adapted four scales devised by Matthews and Barnes-Farrell (2010) to assess these constructs in university students. The 4-item *Work Flexibility-Ability Scale* (e.g., “I am able to arrive and depart from work when I want in order to meet my *study* [original: *family and my personal life*] responsibilities”). Original α was 0.84; current = 0.81. The 4-item *Work Flexibility-Willingness Scale* (“I am willing to take an extended lunch break so that I can deal with *study* responsibilities [original: *relating to my family and personal life*]). Original α was 0.68; current = 0.71. The 5-item *Family Flexibility Scale* (“If the need arose, I could work late without affecting my *university or study* [original: *family and personal*] responsibilities”). Original α was 0.72, current = 0.80. The 6-item *Family Flexibility-Willingness Scale* (“I am willing to change *university or study* plans [original: *my friends’ and family*] so that I can *go to work* [original: *finish a job assignment*]”). Original α was 0.75, current = 0.81. Matthews and Barnes-Farrell provided support for validity by finding expected associations with work-to-nonwork conflict, and work and nonwork centrality.

*Job demands*. The 9-item Psychological Job Demands Scale from the Job Content Questionnaire (Karasek et al., 1998) assesses psychological strain from paid work (e.g., “I am required to work fast” and “I have to work hard”). Participants answered items using a 5-point frequency format (1 *rarely* to 5 *very often*). Niedhammer (2002) found α = 0.77 and supported validity by finding negative relationships with social support. Our α was 0.85.

*Study demands*. The 6-item Role Overload Scale (Thiagarajan et al., 2006), originally developed to examine parent role demands, was adapted to study demands (e.g., “I have to do things *for university* that I do not really have time and energy for”). The authors reported α = 0.87 and supported validity by finding expected correlations with hours worked. Our α = 0.88.

*Work-study conflict*. The 5-item Work-School Conflict Scale (Butler, 2007) was adapted for use with university students (e.g., “Because of my job, I go to *university* tired”). Creed et al. (2015) reported alpha of 0.82 with university students and supported validity by finding positive associations with time, strain, and behaviour-based demands. Our α was 0.87.

*Study burnout*. We adapted Kristensen et al.’s (2005) 7-item Work-Related Subscale from the Copenhagen Burnout Inventory to assess how students felt as a result of their study demands (e.g., “Do you feel worn out at the end of your *study day* [original: *working day*]?”; 5-point response from 1 *rarely* to 5 *very often*). This measure has been adapted previously for use with college students and found to have good reliability and support for validity (*cf.* Marôco and Campos, 2012). Kristensen et al. reported an alpha of 0.87 for the original scale and supported validity by finding negative associations with several health measures. Our α = 0.89.

*Future employability*. A 6-item version of the Perceived Future Employability Scale (Gunawan et al., 2019) was used to assessed confidence of gaining employment when students’ education was completed. We used the 6 highest loading items from the exploratory factor analysis reported by the authors for their 24-item full scale (e.g., “When I complete my studies, I will have gained the knowledge required to get the job I want”). Gunawan et al. (2019) reported an α of 0.95 and supported validity by finding positive correlations with career ambition and university commitment. Our α for the abbreviated scale was 0.87.

### 2.3. Procedure

The study was approved by ethics’ committees of both participating universities. Students were contacted *via* their course websites and provided with a link to an online survey. Participation was voluntary and anonymous, and for their time and effort, students could opt to enter a prize draw for a $50 store voucher. They were advised on the information sheet that submitting the completed questionnaire would indicate their informed consent.

Before conducting the LPA, we performed a CFA to test the construct validity of the scales. We assessed a 6-factor model using all the scales used for the LPA (i.e., work and study congruence, work flexibility-ability and -willingness, and study flexibility-ability and -willingness). For the CFA, we constructed item parcels as these generate fewer and more stable estimates, more parsimonious models to be interpreted, and reduce the possibility of violating assumptions of normality (Little et al., 2013). Parcels were generated by conducting separate exploratory factor analyses for each scale, ordering the items by factor loading, and then assigning a mixture of high- and low-loading items to each parcel (Hau and Marsh, 2004). This model generated a good fit (*cf.* Hair et al., 2010), *χ*^2^(39) = 109.51, *p* < 0.001, *χ*^2^/df = 2.81, CFI = 0.98, and RMSEA = 0.05, which supported proceeding with the LPA.

LPA identifies latent population sub-groups based on scores on predetermined measures of interest, which can be person- or environmental-based. Using latent factor scores, several profiles are generated and these are then assessed to identify the best fitting version (Nylund et al., 2007; Spurk et al., 2020). We used the *tidy*LPA package in R (V4.0.3) with maximum likelihood with robust standard errors (MLR), as this provides more accurate estimates and improves the reliability of results (Nylund et al., 2007). For fit statistics, we consulted Akaike’s Information Criterion (AIC), the Bayesian Information Criterion (BIC), and the sample-size adjusted BIC (SABIC); lower scores for all indicate a better fit (Spurk et al., 2020; Zhang et al., 2021). We conducted Bootstrap Likelihood Ratio Tests (BLRT; w), which assess whether an LPA profile (model *k*) is a good fit compared to whether an alternative model (model *k* + 1) is required; fit is indicated when *k* + 1 is not significant (Spurk et al., 2020). We also assessed entropy (or how distinct profiles are from one another), where higher entropy levels indicate a better fit (ideally >0.60; Jung and Wickrama, 2008).

Last, after identifying the best fitting profiles, we tested, using SPSS (V27), whether these profiles differed on a set of outcome variables that included job demands, study demands, work-study conflict, study burnout, and perceived future employability.

Assumptions for all statistical procedures were assessed and met requirements.

## 3. Results

### 3.1. Relationships among LPA variables

From the correlation matrix, all variables (work and study congruence, flexibility-ability, and flexibility-willingness) were associated weakly to moderately with one another (|*r*| range = 0.13 to 0.39), except for study flexibility-willingness, which was correlated with work flexibility-willingness and study flexibility-ability only, and study flexibility-ability, which was not correlated with work-flexibility-willingness. Correlations with demographic questions ranged from |*r*| 0.01 to 0.25 (see Table 1).

**Table 1:** Summary data and bivariate correlations for LPA and demographic variables; *N* = 808.

Variable	*M*	*SD*	1	2	3	4	5	6
1. Work congruence	18.46	4.32	–					
2. Study congruence	19.51	3.25	0.20***	–				
3. Work flexibility-ability	11.18	4.70	0.37***	0.13***	–			
4. Work flexibility-willingness	15.29	3.87	0.15***	0.13***	0.39***	–		
5. Study flexibility-ability	17.11	4.98	0.18***	0.22***	0.19***	−0.03	–	
6. Study flexibility-willingness	13.67	4.35	−0.03	−0.02	−0.02	−0.18***	0.40***	–
7. Age (years)	19.63	2.24	−0.10**	−0.01	−0.05	−0.06	−0.07*	0.08*
8. Gender^a^	–	–	0.03	−0.05	0.14***	0.07*	0.01	−0.05
9. Hours worked per week	18.72	9.55	−0.22***	0.05	−0.05	−0.07*	−0.01	0.25***
10. Financial situation	1.98	0.82	−0.11**	−0.12**	−0.14***	−0.04	−0.11**	0.04

a 0 = female, 1 = male; *p < 0.05, **p < 0.01, ***p < 0.001.

### 3.2. Latent profile analysis

First, based on Mahalanobis Distance, we deleted four multivariate cases to reduce the possibility that outliers would distort the profiles generated (Spurk et al., 2020). After this, we specified a series of LPA models, varying from one to five profiles. These models, along with their fit statistics, are reported in Table 2. There was a decline in AIC, BIC, and SABIC statistics until the 4-profile model, after which there was an increase. Entropy was highest for the 4-profile solution, and BLRT for this solution was significantly different from the 5-profile model. Based on this, we accepted the 4-profile model.

**Table 2:** Results of latent profile analysis for 1- to 5-profile models (*N* = 804).

Profiles	LL	AIC	BIC	SABIC	BLRT *p*	Entropy
1	−6567.33	13188.66	13315.28	13229.54	–	1.00
2	−6543.48	13154.97	13314.41	13206.44	0.01	0.67
3	−6482.47	13046.93	13239.21	13109.01	0.01	0.71
4	−6456.28	**13008.57**	**13233.67**	**13081.24**	**0.01**	**0.73**
5	−6474.87	13059.74	13317.67	13143.02	0.75	0.48

LL = model log likelihood; AIC = Akaike’s Information Criterion; BIC = Bayesian Information Criterion; SABIC = sample-size adjusted BIC; BLRT *p* = significance level for Bootstrap Likelihood Ratio Test. Boldface indicates best fit.

**Figure 1:**
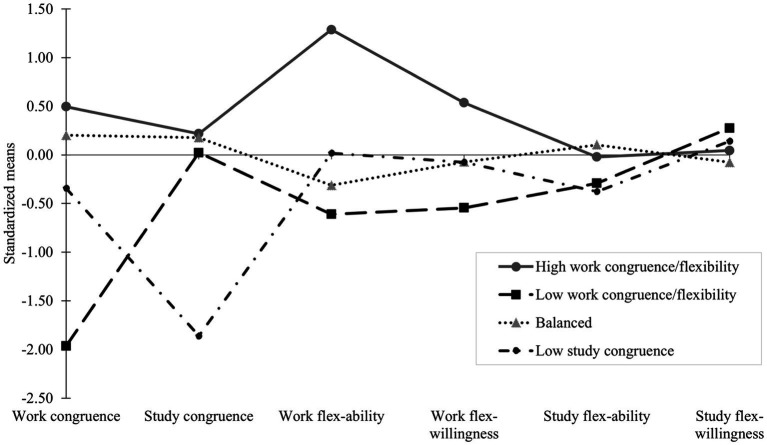
The 4-profile model.

The 4-profile model is reported in Figure 1, with standardized scores indicating *SD* units. We followed recommendations by Gustafsson et al. (2018) and treated values greater than ±0.50 *SD* as indicating a meaningful difference from the mean. Profile 1 contained 17.5% of students who were characterised by higher levels of work congruence (+0.50 *SD*), work flexibility-ability (+1.29 *SD*), and work flexibility-willingness (+0.54 *SD*), and average levels of study congruence (+0.22 *SD*), study flexibility-ability (−0.02 *SD*) and study flexibility-willingness (+0.05 *SD*). This profile was labelled the *high work congruence/flexibility* group.

Profile 2 (9.7%) was characterised by lower levels of work congruence (−1.96 *SD*), work flexibility-ability (−0.61 *SD*), and work flexibility-willingness (−0.54 *SD*), and average levels of study congruence (+0.02 *SD*), study flexibility-ability (−0.29 *SD*) and study flexibility-willingness (+0.28 *SD*). This was labelled the *low work congruence/ flexibility* group. Profile 3 (the largest group; 65.4%) reflected those with average levels of work (+0.20 *SD*) and study congruence (+0.18 *SD*), work (−0.31 *SD*) and study (+0.10 *SD*) flexibility-ability, and work (−0.07 *SD*) and study (−0.08 *SD*) flexibility-willingness and was labelled the *balanced* group. Last, Profile 4 (7.3%) was characterised by lower levels of study congruence (−1.86 *SD*) and average levels of work congruence (−0.34 *SD*), study (−0.38 *SD*) and work (+0.02 *SD*) flexibility-ability, and study (+0.14 *SD*) and work (−0.08 *SD*) flexibility-willingness. This profile was labelled the *low study congruence* group.

Considering shape similarities/differences, Profiles 2 (*low work congruence/ flexibility*) and 4 (*low study congruence*) had similar profiles, except that Profile 2 was characterised by being low on work congruence (−1.96) and Profile 4 by being low on study congruence (−1.86). Profiles 1 (*high work congruence/work flexibility*) and 3 (*balanced*) were similar, with all average scores except Profile 1 reported higher levels of work flexibility-ability and flexibility-willingness. The four profiles were not differentiated by study flexibility-ability and study flexibility-willingness, with variability driven by the relationship with work (congruence), opportunities for flexibility at work (work flexibility-ability), and personal resources for actioning these opportunities (work flexibility-willingness).

### 3.3. Differences in outcome variables across profiles

We tested differences on the four profiles for the outcomes of job demands, study demands, work-study conflict, study burnout, perceived future employability, and the biographic variables (age, gender, hours worked, financial situation). As the assumption of homogeneity of variances were not met for work-study conflict, burnout, employability, age, hours worked, and financial situation, we used Welch’s ANOVA, which adjusts the degrees of freedom accordingly, when testing these variables. For testing gender differences, we used *χ*^2^ cross-tabulation. From these analyses, we found significant differences among the four profiles on all five outcomes and the demographic variables (see Table 3).

**Table 3:** Differences on outcome and demographic variables by profile.

Outcomes	Profile 1: high work congruence/flexibility (*N* = 141)	Profile 2: low work congruence/flexibility (*N* = 78)	Profile 3: balanced (*N* = 526)	Profile 4: low study congruence (*N* = 59)	Differences among profiles
	M (SD)	M (SD)	M (SD)	M (SD)	
Job demands	29.33 (7.35)	33.85 (7.00)	30.56 (6.83)	30.93 (7.03)	2 > 1, 3, 4***
Study demands	18.62 (6.08)	22.29 (6.43)	18.77 (5.86)	21.34 (5.96)	2, 4 > 1, 3***
Work-study conflict	14.21 (5.29)	19.27 (4.03)	15.41 (4.58)	17.92 (4.44)	2, 4 > 1, 3***
Study burnout	23.21 (7.40)	25.55 (7.43)	23.07 (6.37)	25.81 (5.64)	2, 4 > 1, 3***
Future employability	27.67 (4.95)	26.13 (6.85)	27.36 (5.18)	25.08 (6.34)	1, 3 > 4*
Age	19.74 (2.26)	20.38 (2.47)	19.45 (2.12)	20.03 (2.60)	2 > 3**
Hours worked	18.78 (10.53)	25.28 (10.56)	17.79 (8.62)	18.71 (10.51)	2 > 1, 3, 4***
Financial difficulties	1.84 (0.73)	2.28 (0.95)	1.96 (0.79)	2.12 (0.93)	2 > 1, 3**

Scheffé test used for post-hoc comparisons for job demands and study demands; Welch’s ANOVA and Games-Howell test used for *post-hoc* comparisons for remaining variables. For gender (0 = female, 1 = male), there were disproportionately more females in Profile 2 and fewer in Profiles 1 and 4. *p < 0.05, **p < 0.01, ***p < 0.001.

Profile 2 (*low work congruence/flexibility*) reported higher job demands than the other profiles, *F*(3, 800) = 7.26, *p* < 0.001. Profiles 2 (*low work congruence/flexibility*) and 4 (*low study congruence*) had higher study demands, *F*(3, 800) = 10.83, *p* < 0.001; work-study conflict, *F*(3, 165.98) = 28.72, *p* < 0.001, and burnout, *F*(3, 163.30) = 6.09, *p* < 0.001, than Profiles 1 (*high work congruence/flexibility*) and 3 (*balanced*). Last, Profiles 1 and 3 reported higher employability than Profile 4 (*low study congruence*), *F*(3, 157.42) = 3.37, *p* = 0.02.

For the biographic variables, there was little variability among profiles for age (likely due to sample age range restriction of 17–25 years), although Profile 2 (*low work congruence/ flexibility*) was older than Profile 3 (*balanced*), *F*(3, 157.90) = 4.18, *p* = 0.007. Profile 2 also worked more hours per week than all other Profiles, *F*(3, 155.90) = 11.83, *p* < 0.001, and reported more financial difficulties than Profiles 1 and 3, *F*(3, 157.95) = 4.54, *p* = 0.004. There were more female than male students in Profile 2 (*low work congruence/flexibility*; *n* = 67 vs. 59) and fewer female students in Profiles 1 (*high work congruence/ flexibility; n* = 93 vs. 107) and 4 (*low study congruence; n* = 38 vs. 45), *χ*^2^ (3, *N* = 803) = 18.08, *p* < 0.001.

## 4. Discussion

This study was informed by the interactionist/fit (Muchinsky and Monahan, 1987; Van Vianen, 2018) and boundary theories (Kreiner et al., 2009) and contributed novel information on how different groups of students perceived their boundary management between the work and study domains. We sought to identify if there were different profiles based on boundary congruence and flexibility, constructs that represented students’ capacity and willingness to manage the work-study role interface in the context of situational constraints (*H1*) and whether the different profiles differed on work and study demands, role conflict, burnout, and future employability (*H2*). Supporting *H1*, four differentiated profiles were identified: *high work congruence and flexibility* (Profile 1; 17.5%), *low work congruence and flexibility* (Profile 2; 9.7%), *balanced* (Profile 3; 65.4%), and *low study congruence* (Profile 4; 7.5%). Supporting *H2*, these profiles differed on levels of the five study, work, and career measures.

The four profiles were differentiated by role congruence and work flexibility, but not by study flexibility-ability (i.e., student’s *capacity* to be flexible about study commitments) or study flexibility-willingness (i.e., *willingness* to be flexible about study commitments). Despite an increase in flexible learning in recent years, especially since the global pandemic, university students are still restricted when it comes to much of their university life and must make semester-wide study commitments. For example, they have restricted laboratory class times, assignments are expected to be submitted at set times, and exams typically are scheduled for one sitting only, even if this is online to increase flexibility of access (Stone et al., 2019). Additionally, for most working students, study is seen as the main role and work as secondary, and the primary commitment is likely to be to study even though many must work to survive (Butler, 2007). Thus, all students might have had similar perceptions that they have study commitments that they have little opportunity to modify and might not want to modify as they give priority to their study. Both of which might account for the low variability across the four profiles on study flexibility-ability and flexibility-willingness.

Turning to the individual profiles, Profile 3 (i.e., *balanced*) can be considered the reference group as it contained the largest number of students (65.4%). This group reported near-average means (< ±0.05 *SD*) on all boundary congruence and boundary management variables. Profile 1, which was above average (> 0.50 SD above the mean) on work congruence, flex-ability, and flex-willingness (17.5%), was the only group that held above average views of their workplace, perceiving both supportive and flexible working arrangements that were facilitative of study commitments. Profile 2 (i.e., *low work congruence/flexibility*; 9.7%), reported the poorest workplace conditions vis-à-vis study, with the lowest levels of support for study and workplace flexibility, either ability or willingness. Last, Profile 4 (i.e., *low study congruence*; 7.3%), which was the smallest group, reported the most difficulty in the study domain, perceiving that the university did not offer supportive arrangements to facilitate their work and study responsibilities.

Profile 3 (*balanced*; 65.4%) can be considered those students whose work and study role boundaries were structured in such a way as to allow them to adequately manage their work and study responsibilities. This boundary structuring is likely to result from both employer commitments (e.g., to support flexibility to enable student workers to meet study demands) and student effort (e.g., negotiating work flexibility, being willing to accept that flexibility, and rejecting employment that cannot be flexible). Supporting the notion that these students had role boundaries structured to enable management of both work and study, this group reported lower levels of study demands, work-study conflict, and burnout, and higher levels of perceived future employability than Profiles 2 and 4 that had lower work and study congruence, respectively, and lower levels of job demands than Profile 2.

Profile 1 students (*high work congruence and flexibility*; 9.7%) were distinguished by holding positive views of their workplace. They perceived that the workplace arrangements supported their need to manage study (i.e., congruence) and provided high levels of flexibility, which they felt willing to utilise. As a group, similar to the *balanced* group, they reported lower levels of job and study demands, less work-study conflict and study burnout, and more optimism regarding their future employability than Profiles 2 and 4. These results point to benefits for those who are able to structure their work roles in such a way as to generate support from supervisors and co-workers and build in flexible working arrangements. We do not know whether these arrangements were fortuitous or whether the students managed their boundary arrangements to suit. Future research needs to assess this, as determining ways to increase workplace support and flexibility is likely to bring benefits. Consistent with this, previous research has shown that role management by working students was related to better university adaptation (Swanson et al., 2006), higher wellbeing (Chu et al., 2021b), and a more optimistic view of the future (Chu et al., 2021a).

While we demonstrated that average to above average work congruence and flexibility (i.e., Profiles 3 and 1) had the most positive scores on the outcome variables, it will be important now to explore the personal qualities and skills these groups bring to managing work and study boundaries, and whether these qualities and skills can be enhanced and/or developed in students in the other groups that did not have such positive profiles and outcomes. Negotiating skills, for example, might be higher in those students in Profiles 1 and 3, and, if this is the case, training in negotiation, which can be developed or trained successfully in university students (McGuire et al., 2020), could be added to interventions for students who struggle to manage their work and study boundaries.

Students from Profile 2 (*low work congruence/flexibility*; 9.7%) reported the poorest workplace conditions vis-à-vis study. They had the lowest levels of workplace support for study and flexibility. Thus, this group perceived little support for or opportunity to generate workplace flexibility to suit their study, and little preparedness to negotiate more flexible arrangements. Consistent with this, this group reported the highest level of job demands and, along with the low study congruence Profile 4 group, higher levels of study demands, work-study conflict, and burnout than the better placed Profiles 1 and 3. Thus, the lack of workplace congruence and flexibility was reflected in perceptions of higher demands in both the work and study domains, conflict between the domains, and poorer wellbeing.

These students (Profile 2) might be unfortunate to find themselves having to accept work with employers who offer little flexibility, or these poor outcomes might be the result of student characteristics that limit their boundary management negotiations, or a combination of both environmental challenges and person qualities. Potentially, they then are more likely to have to prioritize work responsibilities over study commitments, to the detriment of their studies. However, we cannot determine from the data we collected whether situational or person qualities are more important or whether this leads students to give primacy to the work domain (e.g., to maintain their work and, thus, income) and future studies need to tease this out. Additionally, despite perceiving greater domain demands and conflict and reporting higher levels of burnout, these students did not perceive poorer employability in their future. Perhaps they think that if they can survive in work with little support and flexibility, they will be able to do well in their future post-graduation employment.

Profile 4 (*low study congruence*; 7.5%), the smallest group, was characterised by reporting the most difficulty with the study domain (i.e., that the university did not offer supportive arrangements to facilitate both work and study responsibilities). This group exhibited similarly negative scores on the outcome variables as the group that reported the poorest workplace conditions (i.e., Profile 2). Profile 4 students perceived higher study demands, role conflict, and study burnout, and less optimism for their future employability than Profiles 1 and 3 that were average or above on the workplace support factors. The evidence here for Profile 4 (poor study domain perceptions) and Profile 2 (poor workplace domain perceptions) suggests that difficulties in either domain is associated with poorer outcomes for students, and what is required is for students’ needs to be met in both areas.

The various groups differed on the outcome variables in expected ways (e.g., Profile 1 with high work congruence and flexibility scored more positively), which supported construct validity of the profiles. Also supporting validity, the four groups showed consistent internal associations. For example, Profile 1, with higher work congruence, also reported higher work flexibility-ability and flexibility-willingness; while Profile 4, with lower work congruence, reported lower levels of work flexibility-ability and flexibility-willingness. These linkages are consistent with perceived boundary congruence (i.e., the attained fit between work and study roles) being associated with perceptions that there are opportunities for the individual to make changes at work and that a willingness to negotiate or make these changes would be seen as acceptable by the employer (Kreiner et al., 2009; Eastgate et al., 2021).

Accordingly, for working students to improve their work-study fit, and thereby allow them to cope better with their competing roles, they should develop skills that enhance their work-study flexibility and congruence. For example, being prepared to raise, and act on, changing working times during high study demand periods, such as before university exams, and negotiating with their universities for increased flexibility, such as increasing flexible access to learning options (e.g., for lectures and laboratory work) to facilitate work, is likely to have broad positive outcomes for functioning in both roles. Our results show that to reap these positive benefits, flexibility and congruence do not need to be more than adequate as Profiles 3 and 1 did not differ significantly on these outcomes, despite Profile 1 have higher work congruence and flexibility than Profile 3.

The results from the study are consistent with fit and boundary congruence theory propositions that structuring the interface between roles to suit the individual and involved others, thereby generating a better fit, is beneficial for the individual. Students in Profile 1 (*high work congruence and flexibility*; 17.5%) and Profile 3 (*balanced*; 65.4%), comprising 82.9% of the sample, met or exceeded these requirements and profited from these arrangements: perceiving fewer job and study demands, less work-study conflict and burnout, and better employability outcomes. In contrast, Profile 2 (*low work congruence/flexibility*; 9.7%) and Profile 4 (*low study congruence*; 7.5%), totalling 17% of the sample, reported work and study boundary difficulties, and experienced higher work and study demands, greater work-study conflict, more burnout, and lower levels of employability optimism.

Last, examining differences on the demographic variables provided some explanation for the differences in profiles and suggested that person and contextual affordances should be considered when determining the needs of these different groups. The stand-out was Profile 2 (*low work congruence/flexibility*; 9.7%), which included disproportionately more female students than expected by chance, worked longer hours, reported more financial difficulty (than Profiles 1 and 3), and were older (than Profile 3). This suggested that older female students, who were more financially strained and relied more on their work for income, might be experiencing more challenges when negotiating their needs at work to meet study responsibilities. This is consistent with the broader work-life balance literature that has shown that women have more domestic duties (Drummond et al., 2017) and have less access to government and family financial support (Carreira and Lopes, 2021). Thus, facilitating the functioning of students in this Profile would need to include strategies that seek to redress barriers typically confronted by older female students, such as discrimination in the workforce, as well as developing boundary management skills.

### 4.1. Limitations

First, our study was cross-sectional, and while we drew on widely applied theories of person-environment fit and role boundary congruence, and empirical research has supported that positive boundary experiences generate positive outcomes (Chen et al., 2009; Van Vianen, 2018), we cannot confirm causal relationships, for example, that greater work flexibility-ability and flexibility-willingness lead to more positive future occupational expectations. Studies that collect data over time are needed before strong causal statements can be made. Now that we have shown that different student groups exist based on role boundary management variables, it will be important to clarify this directionality. Longitudinal studies also are needed to test whether the student boundary management strategies are stable over time, and if there are changes, what causes them. Understanding, for example, what experiences enable students to improve their capacity to gain flexibility in the workplace and to act on it, will be important for informing interventions.

Second, future profile analyses might include more direct measures of the integration/segmentation of roles, as this construct plays an important role in boundary management theory (Kreiner, 2006). We assessed the construct indirectly (e.g., boundary flexibility-willingness reflects students’ boundary preferences), but including a direct measure might clarify the extent to which role integration/segmentation preferences and actions differentiate students. Also, our sample contained disproportionately more female than male students. While the bivariate correlations between gender and the predictor variables were trivial, we did find an over-representation of females in Profile 2 (*low work congruence/flexibility*) and under-representations in Profiles 1 (*high work congruence/ flexibility*) and 4 (*low study congruence*), suggesting that females might struggle more with work than males, but this needs to be confirmed in studies with a more equal gender balance.

Third, we focused on a small group of outcomes, and other profile differences need to be assessed. For example, our focus was on work-study conflict reduction as a profile correlate, and it will be important to examine ways by which students can manage their boundaries to produce positive “spill-over effects” (Brough and O’Driscoll, 2015), such as work-to-study enrichment and facilitation. Qualitative studies would be helpful here as well.

Last, we examined a small group of contextual factors, largely person-based demographic differences. Future studies need to assess other person and background factors, such as workplace and family support and levels of individual agency (e.g., proactivity), which have been suggested as being important in role congruence (Chu et al., 2021a).

## 5. Conclusion

We demonstrated the value of deploying a person-centred approach to generate different profiles of working students based on selected boundary management variables. We confirmed that different student groupings could be differentiated from one another, and that these groups differed on a range of important study, work, and career-related variables. Our results suggest that different groups also will require different types of support and intervention, and that treating working students as one homogenous grouping will be less effective for students, and not in the best interest of educational institutions and employers.

## Data availability statement

The raw data supporting the conclusions of this article will be made available by the authors, without undue reservation.

## Ethics statement

The study was reviewed and approved by both Griffith University Human Research Ethics Committee and University of Southern Queensland Human Research Ethics Committee. Participation in the study was voluntary and anonymous, and submitting a completed questionnaire was taken as an indication of informed consent.

## Author contributions

PC, MH, AB, MM, and PB contributed to conception and design of the study. SW and LE organized and managed the databases. SK performed the statistical analysis. PC wrote the first draft of the manuscript. All authors contributed to the article and approved the submitted version.

## Funding

This project was funded by the Australian Research Council (grant: DP180100930).

## Conflict of interest

The authors declare that the research was conducted in the absence of any commercial or financial relationships that could be construed as a potential conflict of interest.

## Publisher’s note

All claims expressed in this article are solely those of the authors and do not necessarily represent those of their affiliated organizations, or those of the publisher, the editors and the reviewers. Any product that may be evaluated in this article, or claim that may be made by its manufacturer, is not guaranteed or endorsed by the publisher.
